# Internet addiction and suicidal ideation among Chinese college students: the mediating role of psychotic-like experiences

**DOI:** 10.3389/fpubh.2023.1276496

**Published:** 2023-09-28

**Authors:** Meng Kang, Bingna Xu, Chunping Chen, Dongfang Wang

**Affiliations:** ^1^Graduate Institute for Taiwan Studies, Xiamen University, Xiamen, China; ^2^Institute of Education, Xiamen University, Xiamen, China; ^3^School of Psychology, Centre for Studies of Psychological Applications, Guangdong Key Laboratory of Mental Health and Cognitive Science, Ministry of Education Key Laboratory of Brain Cognition and Educational Science, South China Normal University, Guangzhou, China

**Keywords:** Internet addiction, psychotic-like experiences, suicidal ideation, college students, mediating role

## Abstract

**Background:**

Individuals with Internet addiction (IA) are at significant risk of suicide-related behaviors. This study aimed to investigate the relationships among IA, psychotic-like experiences (PLEs), and suicidal ideation (SI) among college students.

**Methods:**

A total of 5,366 college students (34.4% male, mean age 20.02 years) were assessed using the self-compiled sociodemographic questionnaires, Revised Chinese Internet Addiction Scale (CIAS-R), 15-item Positive subscale of the Community Assessment of Psychic Experiences (CAPE-P15), Self-rating Idea of Suicide Scale (SIOSS), and 2-item Patient Health Questionnaire (PHQ-2).

**Results:**

The prevalence of IA and SI were 9.3 and 12.1% among Chinese college students, respectively. There were direct effects of IA and PLEs on SI. The total effect of IA on SI was 0.18 (*p* < 0.001). PLEs mediated the relationship between IA and SI (Indirect effect = 0.07).

**Conclusion:**

IA had both direct and indirect effects on SI. These findings enable us to elucidate the mechanism of how IA influences individual SI, which can provide vital information for developing and implementing targeted interventions and strategies to alleviate SI among Chinese college students.

## 1. Introduction

Nowadays, the Internet has become an essential part of people’s lives. As Internet use sharply increases, the problem of Internet addiction (IA) gradually emerges and becomes a hot-debated issue. IA refers to a problematic behavior related to excessive and uncontrollable Internet use (1). In a recent literature review, Pan and colleagues reported that the median prevalence of IA was 7.02% among the general population, and it increased over time (2). The Internet has gained tremendous popularity among college students. Compared with the general population, college students have easier Internet access and more substantial incentives for Internet use. However, college students lack monitoring and obtain more freedom of choice, making them more fragile and vulnerable to IA (3). Prior studies also supported that university life increased the risk of IA (4, 5). Results from the recent meta-analysis indicated a high level of IA (31.51%) among Iranian college students (6). Joseph et al. summarized that the prevalence of IA among Indian college students was approximately 19.9% (7). In China, one meta-analysis including 26 studies documented that the pooled prevalence rate of IA was 11% among Chinese college students (8). Chi et al. have found that 15.2% of college students have IA (9), while a rate reported as 7.9% in Shen et al.’s study (10). It seems that IA prevalence varies due to culture, measures, cut-offs, and definitions of IA across previous studies.

Previous studies showed that IA was associated with academic failure (11), social isolation (12), psychological symptoms (e.g., depression and anxiety) (13), and risky behaviors (e.g., self-harm and suicidality) (14). Among these factors, suicidality has attracted particular attention due to its devastating consequences (15). Suicide is considered as the second leading cause of death among adolescence and young adulthood worldwide (16). While in China, suicide is the leading cause of death in persons aged 15–34 years (17), which accounts for 19% of all deaths (18). In a literature review, Mortier and colleagues reported a pooled prevalence of lifetime and 12-month suicidal attempts among college students were 3.2 and 1.2%, respectively (19). Notably, suicidal ideation (SI) is one of the strongest predictors of eventual suicidal behavior, and undoubtedly, investigation of SI could contribute to the early identification of adolescents who may be at risk for suicide (20). One large-scale cross-sectional survey (*N* = 136,266) has shown that adolescents with IA had a higher prevalence of SI than those without IA (21). However, the relationship between IA and SI (22–24) and the specific mechanism remain unknown.

PLEs are widely conceptualized as the subthreshold psychotic symptomatology among the general population and have also been interpreted as a resemblance of positive symptoms of psychosis in the absence of a full-blown psychotic disorder (25, 26). Based on the psychosis proneness-persistence-impairment model (27), PLEs are influenced by a combination of genetic factors and environmental risks. IA may be an environmental stressor in the etiology of PLEs. For instance, IA triggers a range of unhealthy lifestyles (e.g., staying up late (28)) that may increase the risk of PLEs (29). Few studies have demonstrated that IA was significantly associated with PLEs among adolescents and young adults (30, 31). Moreover, PLEs serve as a robust risk factor for SI. One survey of 8,096 Korean adolescents aged 14–19 years found that higher PLEs are associated with stronger SI (32). According to Yates et al.’ report, individuals with PLEs may be twice as likely to report SI (33). These findings suggest that increased IA is associated with greater PLEs, and PLEs can predict SI. Thus, PLEs are regarded as an underlying mediator between IA and SI.

This study investigated the mediating role of PLEs on the IA-SI relationship. We hypothesized that (a) IA and PLEs have a positive effect SI among college students; (b) PLEs mediate the IA-SI relationship. Given that depression is strongly associated with IA (34), PLEs (35), and SI (36), consequently, the current study aimed to validate the above hypotheses after controlling for individual depression.

## 2. Materials and methods

### 2.1. Participants

Using a cross-sectional design, eight universities/colleges in China [Guangxi Province (Southern China: 2 universities/colleges), Hainan Province (Southern China: 1 university/college), Gansu Province (Northwestern China: 1 university/college), Shandong Province (Eastern China: 1 university/college), Hunan Province (Central China: 2 universities/colleges), and Guangdong Province (Southern China: 1 university/college)] were selected by convenience sampling method during July 2023. The study was conducted through the “Questionnaire Star” system. College students used their cell phones to scan the Quick Response (QR) code to access the questionnaire page and complete the survey. This survey is anonymous, and participants can stop or withdraw at any time if they feel uncomfortable during the survey. Finally, 5,824 college students participated in the web-based survey and provided complete data on all measures. In order to improve the quality of data, exclusion criteria for participation included: (a) time to complete the survey <5 min; (b) have current significant mental disorders that were identified by self-reported; (3) scores above 4 on the dishonesty subscale of the Self-rating Idea of Suicide Scale (SIOSS) (37, 38). Among these participants, 458 college students were subsequently removed, leaving 5,366 with valid data entry for further analyses.

### 2.2. Measures

#### 2.2.1. Sociodemographic variables

The sociodemographic variables of the participants included age, sex, grade, ethnicity, single-child status, parental marital status, family income, and parents’ educational level.

#### 2.2.2. IA

The Revised Chinese Internet Addiction Scale (CIAS-R) was used to assess IA (39). It consists of 19 items, clustering into four dimensions: compulsive use of the Internet or withdrawal symptoms, tolerance symptoms, interpersonal and health-related problems, and time management problems. Each item was rated on a four-point Likert scale, from 1 (‘complete inconformity’) to 4 (‘complete conformity’). The higher the total score, the greater the level of IA. According to previous studies (10, 40), the CIAS-R has satisfactory reliability and validity among Chinese college students, and a cut-off total score of 53 has been suggested to identify probable IA. In our study, Cronbach’s α was 0.98.

#### 2.2.3. PLEs

The 15-item Positive subscale of the Community Assessment of Psychic Experiences (CAPE = P15) was adopted to measure PLEs (41). The scale includes three dimensions, namely persecutory ideation, bizarre experiences, and perceptual abnormalities. Each item was rated within a time frame of the last month on a four-point Likert scale, from 1 (‘never’) to 4 (‘nearly always’), with higher scores reflecting more frequent PLEs. The Chinese version of CAPE-P15 has demonstrated acceptable reliability and construct validity among college students (42). Participants were regarded to have genuine PLEs in the past month when they scored ≥ 1.57 on an average score of items in CAPE-P15 (43). In our study, Cronbach’s α was 0.96.

#### 2.2.4. SI

The Self-rating Idea of Suicide Scale (SIOSS) was utilized to assess SI (37). It comprises 26 items within four dimensions, which are despair, pessimism, sleep, and concealment. Each item was answered on a dichotomous scale from 0 (no) to 1 (yes). We added the first three subscales (despair, pessimism, and sleep) to generate a total score of SI, with higher scores indicating stronger SI. Participants are considered to have SI when their score reaches or exceeds 12 (37). Meanwhile, the test is invalid, if the concealment factor is ≥4 (37). The SIOSS has satisfactory psychometric properties among Chinese college students (38, 44). In our study, Cronbach’s α was 0.88.

#### 2.2.5. Depression

The 2-item Patient Health Questionnaire (PHQ-2) was employed for screening depressive symptoms over the past 2 weeks (45). Each item was rated on a four-point Likert scale, from 0 (‘not at all’) to 3 (‘nearly every day’), with higher total scores indicating more significant depressive symptoms. A total score ≥ 3 refers to the positive result of depression. The PHQ-2 is widely used among Chinese college students with good reliability and validity (46, 47). In our study, Cronbach’s α was 0.80.

### 2.3. Ethics statement

The studies involving human participants were reviewed and approved by the Ethics Board of the South China Normal University, China (SCNU-PSY-2022-235). All participants who volunteered to participate were informed of the purpose, process, benefits, and risks.

### 2.4. Statistical analysis

Data were analyzed through IBM SPSS Statistics Version 23.0. The sociodemographic characteristics and variables were described with frequency (proportion) for categorical variables and mean (standard deviation, SD) for continuous variables. The *χ*^2^ tests and Mann–Whitney U tests were used to compare differences in categorical and continuous variables between the IA and non-IA groups, respectively. The hierarchical multiple logistic regression was conducted to explore the associations between IA and SI. In step 1, the model was unadjusted by setting SI as the dependent variable and IA as the independent variable. In step 2, we made adjustments for all sociodemographic variables. In step 3, school PLEs was added, and depression was added in the last step. The results were demonstrated with odds ratios (ORs) and their 95% confidence intervals (CIs). Spearman correlation analysis examined the association among IA, PLEs, SI, and depressive symptoms. The mediating hypothesis was tested via PROCESS (48). Bootstrap analysis was conducted with 5,000 iterations yielding 95% confidence intervals estimating the size of each model’s effects. The mediation effect (model 4) was tested: IA was entered as the predictor, PLEs as the mediator, and SI as the outcome. All sociodemographic variables and depression were included in the analyses as covariates. The same approach examined the mediating role of PLEs in males and females without controlling for sex. Tests of statistical significance were two-sided, and statistical significance was set at α = 0.05.

## 3. Results

### 3.1. Sample characteristics

This study comprised 5,366 college students (mean age: 20.02 ± 1.38 years, 34.4% male). The majority of the students were Ethnicity Han (84.0%). More than half of the students are freshmen (52.1%). Further sample characteristics are presented in Table 1.

**Table 1:** Descriptive statistics of participants [*N* (%)].

Variables		Overall *N* = 5,366	Non-IA 4865 (90.7%)	IA^e^ 501 (9.3%)	χ^2^/*Z*	*p*
Age [year]	Mean(SD)	20.02 (1.38)	20.01 (1.37)	20.11 (1.47)	−0.99	0.321
Sex	Male	1846 (34.4)	1,694 (34.8)	152 (30.3)	4.04	0.048
	Female	3,520 (65.6)	3,171 (65.2)	349 (69.7)		
Grade	Freshman	2,796 (52.1)	2,569 (52.8)	227 (45.3)	18.39	<0.001
	Sophomore	1,484 (27.7)	1,315 (27.0)	169 (33.7)		
	Junior	907 (16.9)	828 (17.0)	79 (15.8)		
	Senior	179 (3.3)	153 (3.2)	26 (5.2)		
Ethnicity	Han^a^	4,507 (84.0)	4,106 (84.4)	401 (80.0)	6.42	0.013
	Others	859 (16.0)	759 (15.6)	100 (20.0)		
Single child status	Yes	898 (16.7)	834 (17.1)	64 (12.8)	6.22	0.012
	No	4,468 (83.3)	4,031 (82.9)	437 (87.2)		
Parental marital status	Married	4,733 (88.2)	4,303 (88.4)	430 (85.8)	3.00	0.094
	Not current married^b^	633 (11.8)	562 (11.6)	71 (14.2)		
Family income (monthly), RMB	<3,000	2,119 (39.5)	1917 (39.4)	202 (40.3)	0.35	0.840
	3,000 ~ 5,000	1742 (32.5)	1,578 (32.4)	164 (32.7)		
	>5,000	1,505 (28.0)	1,370 (28.2)	135 (26.9)		
Father’s education	Junior high school or less	3,715 (69.2)	3,354 (68.9)	361 (72.1)	2.23	0.328
	Senior high school	1,053 (19.6)	966 (19.9)	87 (17.4)		
	College or more	598 (11.1)	545 (11.2)	53 (10.6)		
Mother’s education	Junior high school or less	4,176 (77.8)	3,764 (77.4)	412 (82.2)	6.24	0.044
	Senior high school	728 (13.6)	674 (13.9)	54 (10.8)		
	College or more	462 (8.6)	427 (8.8)	35 (7.0)		
CAPE-P15 score	Mean(SD)	19.06 (6.56)	18.33 (5.48)	26.13 (10.67)	−20.16	<0.001
SIOSS score	Mean(SD)	5.25 (4.62)	4.79 (4.33)	9.74 (4.85)	−21.16	<0.001
PHQ-2 score	Mean(SD)	1.72 (1.30)	1.60 (1.23)	2.89 (1.35)	−21.06	<0.001
PLEs^c^	Yes	976 (18.2)	726 (14.9)	250 (49.9)	373.45	<0.001
SI^d^	Yes	650 (12.1)	466 (9.6)	184 (36.7)	314.45	<0.001

a Han is the major ethnic group in China.

b Not current married included separated, divorced and widowed.

c PLEs calculated using the CAPE-P15, with a clinical cut-off average score of 1.57.

d SI calculated using the SIOSS, with a clinical cut-off total score of 12.

e IA calculated using the CIAS-R, with a clinical cut-off total score of 53.

CAPE-P15, 15-item Positive Subscale of the Community Assessment of Psychic Experiences; SIOSS, Self-rating Idea of Suicide Scale; PLEs, psychotic-like experiences; SI, suicidal ideation; IA, internet addiction.

The prevalence of IA and SI were 9.3 and 12.1% among Chinese college students. The prevalence of IA among males and females were 8.2 and 9.9%, respectively, (*χ*^2^ = 4.04, *p* = 0.048). The prevalence of SI among the male and female were 12.4 and 12.0%, respectively (*χ*^2^ = 0.15, *p* = 0.692). 36.7% of college students with IA reported the presence of SI, which was significantly higher than the rate reported in those without IA (9.6%, *χ*^2^ = 314.45, *p* < 0.001). Students with IA also showed significantly higher severity of PLEs (*Z* = −20.16, *p* < 0.001) and depression (*Z* = −21.06, *p* < 0.001) than those without IA. Table 1 also compares sample characteristics and other study variables between IA and non-IA groups.

### 3.2. Hierarchical regression analyses

Table 2 illustrates the results of hierarchical regression analyses. Participants who reported having IA were more likely to have SI (OR = 5.48; 95% CI = 4.46–6.73). After controlling for all sociodemographic variables, IA was still significantly associated with increased odds of SI (OR = 5.59; 95% CI = 4.54–6.89). Furthermore, this association remained significant after adjusting for PLEs and depression [OR (95% CI) = 3.56 (2.84, 4.45) and 2.34 (1.84, 2.98) in Step 3 and Step 4, respectively].

**Table 2:** Results of hierarchical regression analyses [OR (95%CI)].

Variables		Step 1	Step 2	Step 3	Step 4
Age	–		0.91 (0.83,0.98)^*^	0.92 (0.84,1.00)	0.94 (0.86,1.03)
Sex	Male		Ref.	Ref.	Ref.
	Female		0.87 (0.72,1.04)	0.99 (0.82,1.20)	0.97 (0.80,1.18)
Grade	Freshman		Ref.	Ref.	Ref.
	Sophomore		1.29 (1.04,1.60)^*^	1.28 (1.03,1.60)^*^	1.19 (0.94,1.50)
	Junior		1.40 (1.04,1.88)^*^	1.43 (1.05,1.94)^*^	1.33 (0.97,1.83)
	Senior		1.27 (0.74,2.18)	1.32 (0.77,2.32)	1.14 (0.64,2.04)
Ethnicity	Han^a^		Ref.	Ref.	Ref.
	Others		1.13 (0.90,1.42)	1.13 (0.90,1.43)	1.10 (0.86,1.40)
Single child status	Yes		Ref.	Ref.	Ref.
	No		0.88 (0.69,1.12)	0.82 (0.64,1.05)	0.82 (0.64,1.06)
Parental marital status	Married		Ref.	Ref.	Ref.
	Not current married^b^		1.33 (1.04,1.70)^*^	1.32 (1.03,1.70)^*^	1.21 (0.93,1.58)
Family income (monthly), RMB	<3,000		Ref.	Ref.	Ref.
	3,000 ~ 5,000		0.63 (0.50,0.80)^***^	0.70 (0.56,0.89)^**^	0.73 (0.57,0.93)^*^
	>5,000		0.62 (0.50,0.76)^***^	0.65 (0.52,0.80)^***^	0.67 (0.54,0.84)^***^
Father’s education	Junior high school or less		Ref.	Ref.	Ref.
	Senior high school		0.82 (0.64,1.05)	0.82 (0.64,1.06)	0.87 (0.67,1.14)
	College or more		1.00 (0.71,1.40)	1.04 (0.74,1.48)	1.20 (0.84,1.73)
Mother’s education	Junior high school or less		Ref.	Ref.	Ref.
	Senior high school		1.26 (0.96,1.67)	1.26 (0.95,1.67)	1.23 (0.92,1.65)
	College or more		1.33 (0.91,1.94)	1.26 (0.85,1.86)	1.09 (0.72,1.64)
Depression^a^	Yes				5.18 (4.28,6.27)^***^
PLEs^b^	Yes			4.27 (3.54,5.14)^***^	3.20 (2.63,3.90)^***^
IA^c^	Yes	5.48 (4.46,6.73)^***^	5.59 (4.54,6.89)^***^	3.56 (2.84,4.45)^***^	2.34 (1.84,2.98)^***^

a Depression calculated using the PHQ-2, with a clinical cut-off total score of 3.

b PLEs calculated using the CAPE-P15, with a clinical cut-off average score of 1.57.

c IA calculated using the CIAS-R, with a clinical cut-off total score of 53.

PLEs, psychotic-like experiences; IA, internet addiction; OR, odds ratio; CI, confidence interval. Step 1: unadjusted. Step 2: adjusted for age, sex, grade, ethnicity, single child status, parental marital status, family income (monthly), and parents’ education. Step 3: Model 2 variables + PLEs. Step 4: Model 3 variables + Depression. *p < 0.05; ^**^p < 0.01; ^***^p < 0.001.

### 3.3. The mediating role of PLEs

As shown in Table 3, IA is positively significantly associated with PLEs (*r* = 0.54, *p* < 0.001), SI (*r* = 0.41, *p* < 0.001), and depression (*r* = 0.45, *p* < 0.001).

**Table 3:** Spearman’s correlation coefficients between all study variables [*r*].

	IA	PLEs	SI	Depression
IA	1			
PLEs	0.54	1		
SI	0.41	0.41	1	
Depression	0.45	0.43	0.53	1

IA, internet addiction; PLEs, psychotic-like experiences; SI, suicidal ideation. All *p* < 0.001.

**Figure 1:**
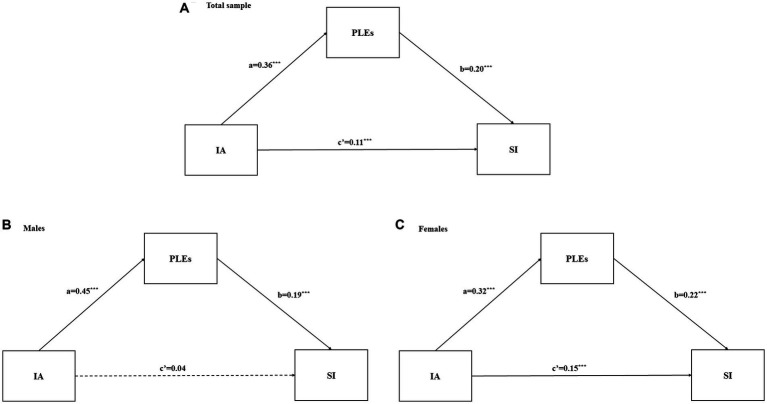
**(A-C)** Path model testing the mediation effect of PLEs in the association between IA and SI. IA, internet addiction; PLEs, psychotic-like experiences; SI, suicidal ideation. ^*^p < 0.05; ^**^p < 0.01; ^***^p < 0.001.

Table 4 described the standardized regression results of mediation to test the effects of IA on SI through the mediator of PLEs. Sociodemographic variables and depression served as covariates in the presented models. The total effect of IA on SI was 0.18 (*p* < 0.001). As shown in Figure 1A, IA had a significant positive effect on both PLEs (*β* = 0.36, *p* < 0.001) and SI (*β* = 0.11, *p* < 0.001). PLEs also positively predicted SI (*β* = 0.20, *p* < 0.001). The bootstrap results with 5,000 resample revealed that IA exerted a significant indirect effect on SI, due to the mediating effect of PLEs (effect = 0.07, 95% CI = 0.06–0.09). Thus, PLEs partially mediated the relationship between IA and SI in the total sample.

**Table 4:** The mediating effect of PLEs on the relationship between IA and SI by sex.

Pathway	Effect	S.E.	95%CI
Total sample			
Total effect (c)	0.18	0.01	0.16, 0.21
Direct effect(c’)	0.11	0.01	0.09, 1.14
Indirect effect (a × b)	0.07	0.01	0.06, 0.09
Males			
Total effect (c)	0.13	0.02	0.08, 0.17
Direct effect(c’)	0.04	0.02	−0.01, 0.09
Indirect effect (a × b)	0.09	0.01	0.06, 0.11
Females			
Total effect (c)	0.22	0.02	0.19, 0.25
Direct effect(c’)	0.15	0.02	0.12, 0.18
Indirect effect (a × b)	0.07	0.01	0.05, 0.08

All continuous variables were standardized before they were entered in the path model. All mediation models adjusted for age, sex, grade, ethnicity, single child status, parental marital status, family income (monthly), parents’ education, and depression.

As shown in Table 4, separate analyses showed that the indirect effects in males were 0.09 (95% CI: 0.06–0.11). The direct effect was not statistically significant (95% CI: −0.01–0.09) after accounting for the effect of confounders factors in males. In comparison, the direct and indirect effects in females were 0.15 (95% CI: 0.12–0.18) and 0.07 (95% CI: 0.05–0.08). Thus, PLEs fully mediated the association between IA and SI in males (Figure 1B), while partially mediating the IA-SI relationship in females (Figure 1C).

## 4. Discussion

This study deepened our understanding of how IA impacts SI through the mediating role of PLEs among Chinese college students. Evidence shows that IA and PLEs were associated with SI, controlling for depression. Meanwhile, PLEs mediated the IA-SI relationship.

In our study, approximately 9.3% of college students reported IA. Shen et al. reported that the prevalence of IA between February to June 2019 was 7.7% among Chinese college students (N = 8,098) with the same scale and cut-off point (10). The increase in this rate can be explained by the COVID-19 pandemic of recent years. As a result of the pandemic lockdown, college students have much greater access to the Internet for entertainment, online courses, and connecting with friends, leading to an increased risk of IA (49). The prevalence rate of IA is slightly higher for female students compared with male students in our study. This is consistent with the results from Deb et al.’s study among 258 medical students in West Bengal (50) and Shen et al.’s study among 8,098 college students in China (40). Contrary findings have been obtained from one meta-analysis based on Chinese college students, indicating the prevalence of males in IA was found to be higher (8). This may be related to females’ increased demand for online social interactions (e.g., WeChat) (51) and online shopping (e.g., Taobao) (52). Among these college students, the self-reported prevalence of SI was 12.1%, slightly lower than the results of a survey of medical students with migraines using the same measure (13.7%) (44). Meanwhile, the SI prevalence did not differ by sex, which aligns with a previous meta-analysis (53).

Compared with those without IA, students with IA had a higher prevalence of SI, which validated our hypothesis. Specifically, students with IA were 5.48 times more likely than other students to develop SI. Extensive works have proved the association between IA and SI (14). For example, Kuang et al. proposed that greater IA increased the likelihood of SI (21). One possible reason is that students may acquire harmful online information about suicide and hold a positive attitude toward suicide, which has been associated with an increased risk of SI (54). Further, in line with previous studies (32, 33), PLEs were significantly associated with higher endorsement of SI in the present study. Echoing Sun et al.’s study, this result indicated that PLEs can be considered as a promising predictor for SI independent of the other psychopathology during the pandemic lowdown (55).

Our observations confirmed our hypothesis, that the direct link between IA and SI is mediated by PLEs after adjusting for sociodemographic variables and depression. This result suggests that IA may increase susceptibility to PLEs. The possible reasons are that individuals who overuse the internet are more likely to develop poor habits that are detrimental to their mental health, such as lack of physical activity (56), irregular diet (57), and reduced sleep duration (29). Meanwhile, people with IA may further isolate others (12), neglect meaningful relationships, lack social support, and feel loneliness, which, in turn, may increase the risk of PLEs among vulnerable groups (58). The finding supports our hypothesized model, suggesting that IA may affect college students’ SI by worsening PLEs. Moreover, our finding also indicated that PLEs fully mediate the association between IA and SI in males, while partially mediating the IA-SI relationship in females. This suggests that PLEs have a greater percentage of mediation for the IA-SI relationship in males. As for females, in addition to PLEs and negative emotions, other factors may influence the relationship between IA and SI, such as insomnia symptoms (24). Previous work also demonstrated that the IA-insomnia symptoms link was greater in females than males (59). Thus, further research is necessary to explore sex differences in the mechanisms of the association between IA and SI.

To our knowledge, this study is the first to examine the specific mediating effect of PLEs in the association between IA and SI among a large sample of college students. These findings underscore that IA should be evaluated in future research and educational and clinical practice. Interventions for SI might benefit from promoting coping strategies to reduce IA. Psychological therapies, such as multi-family group therapy (60), and cognitive behavioral therapy (CBT) (61), have shown significant effectiveness in reducing IA. Moreover, PLEs should be assessed and intervened for college students who have IA, to reduce the risk of SI. Reduction in PLEs can be achieved by promoting psychological resilience (62) or improving sleep quality (63).

However, this study has several limitations that need to be clarified. Firstly, all study variables relied on self-report questionnaires rather than clinical diagnosis, which might cause potential reporting bias and potentially threaten the validity of the findings. Clinical interviews also should be conducted to determine the severity of IA, PLEs, and SI in the future. Secondly, the cross-sectional design of this study also limits the ability to make causal inferences. Further longitudinal studies are therefore necessary to validate the current results. Thirdly, since suicide attempts and behaviors were not included in our study, extended research is expected to further clarify the association between IA and comprehensive suicide indexes. Finally, this study did not consider other critical potential confounders, such as insomnia and adverse life events. Our study also did not examine the majors of college students, although there is evidence that study field may be related to college students’ SI (64). Therefore, examining the role of more extensive risk factors in this association is also warranted.

## 5. Conclusion

In conclusion, this study suggests that PLEs have a mediating effect on the association between IA and SI among Chinese college students. This indicates that college students with IA can be considered a high-risk group for SI, which requires early intervention. When intervening, the effects of PLEs on students’ SI should be comprehensively considered.

## Data availability statement

The original contributions presented in the study are included in the article/supplementary material, further inquiries can be directed to the corresponding author.

## Ethics statement

The studies involving humans were approved by Ethics Board of the South China Normal University. The studies were conducted in accordance with the local legislation and institutional requirements. Written informed consent for participation in this study was provided by the participants’ legal guardians/next of kin.

## Author contributions

MK: Conceptualization, Writing – original draft. BX: Writing – review & editing, Conceptualization, Visualization. CC: Writing – review & editing, Conceptualization. DW: Conceptualization, Data curation, Formal analysis, Project administration, Writing – review & editing.
